# Supplementary material for: Factors associated with sexual violence against reproductive-age women in Ghana: A multilevel mixed-effects analysis
Source: PLoS One. 2024 Oct 3;19(10):e0311682. doi: 10.1371/journal.pone.0311682 (PMC11449305; doi:10.1371/journal.pone.0311682)
Supplement: Attachment — Submitted filename: Review_PONE-D-24-14631.pdf [file pone.0311682.s002.pdf]

Referee Report on Factors associated with sexual violence against reproductive-age women in Ghana: a multilevel mixed-effects analysis

PONE-D-24-14631

June 5, 2024

Summary

This paper presents a statistical analysis of recently released data on sexual violence from the Demographic and Health Survey (DHS) in Ghana. The authors use data on individual, partner, household, and community-level characteristics to examine their associations with incidence of sexual violence among married/co-habiting female respondents in Ghana. The paper is timely in providing this evidence since the data has just been released, but it could use some improvements to help address some methodological as well as conceptual issues. In particular, it could be better situated within the Ghanian context of previous studies exploring *any* kind of violence faced by women. Overall, I found the writing to be well structured and coherent, but lacking in some key areas for clarity. Below are some suggestions that could help the authors in revising the paper.

Major Comments:

- **Motivation for the paper**: The paper starts off well by describing the importance of studying sexual violence. However, it could do with more emphasis on *why* authors are focusing on sexual violence over other types of violence faced by women (and on which data is available as well). This would help strengthen the argument and also streamline later parts of the discussion.

- **Contribution to literature**: As mentioned above, we don't know enough about how this study differs from (or how it adds to) existing literature on sexual (and other types of) violence in Ghana. Presumably, the DHS has previous editions in Ghana, and it would be good to cite this evidence as a starting point. On p.4, line 90, the authors argue for the novelty of the study, but do not speak more to this point. Although I am not an expert on studies of this nature in Ghana, I was able to find a few studies that indeed do contribute to this. For example, Tenkorang et al. 2013 have done this with the 2012 data from DHS Ghana. There is also qualitative work on this domain that could help sharpen the discussion (Apatinga and Tenkorang, 2021). More on this below.

- **Variables**: The variable that is the key dependent variable is not defined very clearly. On p.5, line 116, the authors define it as a percentage, but this would

only be true if the estimation was run at a community level. Since the authors are making use of multi-level logistic regression models, my assumption is that this is likely an error in definition. Later on (line 120) they discuss how the main dependent variable of sexual violence takes a value of 1 if certain criteria are met and 0 otherwise, suggesting that it is indeed not measured in percentage terms. Given that the authors use three variables as conditions to determine whether or not the respondent experienced sexual violence, it might also be worth exploring – as a sensitivity check – what the results look like if they were to make the criteria more or less stringent, or even just using one question as a measure of experience of sexual violence.

- **Community-level variables**: The authors include as part of their multi-level estimates community variables that capture community-level poverty and literacy. First, there is no mention of these beforehand coming from the literature or elsewhere, so it would be good to set up the motivation for why we think community-level is important to explore. Second, it also becomes important to then account for secondary data or any other policy-type variables such as laws related to sexual violence (Bhuwania et al., 2024). One last note on using community-level variables, or aggregating using household or respondent level data – it would be good to have information on how exactly this was done. For example, are these weighted averages, do they include only women who have faced violence, or all women, or all individuals/households? More importantly, is it net-of-own aggregate, so that it excludes the current woman/household from the estimation? These are important methodological concerns that need to be detailed, even if only in a footnote.

- **Statistical model**: The model as it stands is not sufficiently motivated, and by p.6 when a reader lands on it, it is unclear what the basis for this model is. It is also unclear why authors use a non-standard level of statistical significance (20%) to consider variables for further analysis. Again, calling back to the literature on using multi-level mixed effects model to study similar questions would be useful. Further, it might be good to have some info on what the benefit of selecting these models in this step-wise way. What is the benefit of choosing pre-determined variables over, say, using a lasso model or similar to find covariates that maximize the fit (if that is the goal of the model).

- **Discussion**: As with the introduction, the discussion section needs more work to better situate the findings of the model with other studies from Ghana. It would also be good, where appropriate, to mention findings from qualitative studies as well (Cannon et al., 2020; Apatinga and Tenkorang, 2022). This might help bridge some gaps in terms of what the authors find and are able to link to Ghanian context. This is also a good place to talk about any cultural beliefs or social norms around sexual violence in Ghana, since the prevalence is also being discussed. The authors could use some sub-group analysis (instead of multi-level models) to understand how poverty and literacy could vary the levels of sexual violence and their determinants. This is an important part of the story that needs to be included carefully.

I hope that the authors find these comments useful and are able to improve the paper to better situate such studies in the context of the literature of sexual violence in Ghana.

References

Apatinga, G. A. and Tenkorang, E. Y. (2021). Determinants of sexual violence against married women: Qualitative evidence from ghana. *Sexual Abuse*, 33(4):434–454. PMID: 32116110.

Apatinga, G. A. and Tenkorang, E. Y. (2022). Barriers to help-seeking for sexual violence among married or cohabiting women in ghana. *Violence Against Women*, 28(15-16):3844–3864. PMID: 34903095.

Bhuwania, P., Raub, A., Sprague, A., Martin, A., Bose, B., Kidman, R., Nandi, A., Behrman, J. R., and Heymann, J. (2024). Impact of laws prohibiting domestic violence on wasting in early childhood. *PLoS one*, 19(3):e0301224.

Cannon, L. M., Sheridan-Fulton, E. C., Dankyi, R., Seidu, A.-A., Compton, S. D., Odoi, A., Darteh, E. K., and Munro-Kramer, M. L. (2020). Understanding the health-care provider response to sexual violence in ghana: a situational analysis. *PLoS one*, 15(4):e0231644.

Tenkorang, E. Y., Owusu, A. Y., Yeboah, E. H., and Bannerman, R. (2013). Factors influencing domestic and marital violence against women in ghana. *Journal of Family Violence*, 28:771–781.
