# Supplementary material for: Factors associated with sexual violence against reproductive-age women in Ghana: A multilevel mixed-effects analysis
Source: PLoS One. 2024 Oct 3;19(10):e0311682. doi: 10.1371/journal.pone.0311682 (PMC11449305; doi:10.1371/journal.pone.0311682)
Supplement: Attachment — Submitted filename: comments, Negussie, May 22, 2024.docx [file pone.0311682.s001.docx]

Sampling Procedure:

1) **You employed a stratified two-stage cluster sampling design.**

First Stage: Target Clusters Selection

- Target clusters were selected from the sampling frame using probability proportional to size.
- **Clarification on Cluster Units**: The unit of cluster refers to the residency type, such as urban or rural areas. Stratification was based on these conditions to ensure representation across different residency types.

Second Stage: Household Selection

- In the second stage, households (HH) were selected within each chosen cluster.

2) **Variables:**

Dependent Variable: Sexual violence in a lifetime.

- **Recommendation**: Consider using a more recent outcome rather than a lifetime measure. Lifetime measures may encompass a range of determinants and outcomes, making it difficult to isolate specific factors. This should be noted as a limitation.

3) **Analysis Section:**

The data were weighted to account for the sampling weight, primary sampling unit, and strata, ensuring appropriate statistical analysis.

- **Primary Sampling Unit and Strata Variables**: It's beneficial to specify the variables used for the primary sampling unit, strata, and weighting to educate the reader on the methodology.

4) Model Building:

Formulas for the Intraclass Correlation Coefficient (ICC) and Median Odds Ratio (MOR) were used but not explained.

- **Symbols Explanation**: Please provide descriptions for each symbol used in the formulas.

**5. Result session:**

Random Effect and Model Comparison:

- **Placement of Description**: It's recommended to place the description of random effects and model comparisons after assessing the factors associated with sexual violence. The model conclusions on the variance effects related to clustering should follow the findings of the association.

5) **Discussion:**

- **Summary of Findings**: Start by summarizing the key findings of your analysis before discussing them in detail.
- **Comparison with Other Countries**: While comparing the prevalence of sexual violence with other African countries, include specific figures from Ghana for a comprehensive comparison.
- **Time Component in Prevalence**: Clarify whether the prevalence is for a lifetime or a 12-month period. Typically, prevalence is a rate with a time component, so it's important to specify this.

The second paragraph in the discussion and the first paragraph in the conclusion should be revised for clarity:

"In the multivariable mixed-effect binary logistic regression model, variables such as age, women’s decision-making autonomy, husband/partner alcohol consumption, sex of household head, and justification of beating if a wife refuses to have sex with her husband were found to be significant factors associated with sexual violence."

- **Clarification of Variables**: Provide specific directions for each variable:
- **Age:** Specify the age groups associated with increased risk.
- **Women’s decision-making autonomy**: Indicate women with no decision-making autonomy as being more at risk.
- **Husband/partner alcohol consumption**: State that alcohol consumption by the husband/partner increases the risk.
- **Sex of household head**: Specify if the sex of the household head (e.g., male-headed households) is a factor.
- **Justification of beating:** Indicate that the belief in the justification of beating if a wife refuses sex is associated with higher risk.
